# Effectiveness and safety of acupotomy for treating back and/or leg pain in patients with lumbar disc herniation: A study protocol for a multicenter, randomized, controlled, clinical trial

**DOI:** 10.1097/MD.0000000000011951

**Published:** 2018-08-24

**Authors:** Jeong Kyo Jeong, Young Il Kim, Eunseok Kim, Hae Jin Kong, Kwang Sik Yoon, Ju Hyun Jeon, Jae Hui Kang, Hyun Lee, Ojin Kwon, So-Young Jung, Chang-Hyun Han

**Affiliations:** a Department of Acupuncture and Moxibustion Medicine, Daejeon University Dunsan Korean Medicine Hospital, Daejeon; b Department of Acupuncture and Moxibustion Medicine, Daejeon University Cheonan Korean Medicine Hospital, Cheonan; c Clinical Medicine Division, Korea Institute of Oriental Medicine, Daejeon, Republic of Korea.

**Keywords:** acupotomy, back and/or leg pain, lumbar disc herniation, protocol, randomized controlled trial

## Abstract

**Background:**

As the number of patients suffering from back and/or leg pain due to lumbar disc herniation (LDH) is increasing in Korea, conservative treatments for patients with LDH have been spotlighted. Although several studies have been published on the use of acupotomy for the treatment of such patients, most of them are only case studies. Therefore, the purpose of this study is to compare the effectiveness and safety of acupotomy to those for manual acupuncture for the treatment of patients with LDH.

**Methods/design:**

This study is planned as a multicenter, parallel design, randomized, controlled, clinical study. One hundred forty-six patients diagnosed with LDH will be randomly assigned to either the acupotomy group or the manual acupuncture group at a 1:1 ratio. Participants in both groups will receive a total of 4 interventions over 2 weeks. An effectiveness assessment based on the outcomes on the visual analog scale (VAS), Roland Morris Disability Questionnaire (RMDQ), Modified-Modified Schober Test (MMST), EuroQol Five Dimensions (EQ-5D), and Patient Global Impression of Change (PGIC) will be conducted at baseline and at 2, 4, and 6 weeks postrandomization. The primary outcome will be the mean change in the VAS for back and/or leg pain 4 weeks postrandomization. Adverse events will be recorded at every visit.

**Discussion:**

The results of this study will provide evidence for the effectiveness and safety of acupotomy treatment for patients with LDH.

**Trial registration:**

Clinical Research Information Service of South Korea (CRIS- KCT0002824), April 24, 2018.

## Abstract 2

Supplemental Digital Content is available in the text

## 1 Introduction

According to the Health Insurance Statistical Yearbook (2012–2016)^[1–5]^ published by the National Health Insurance Corporation and the Health Insurance Review and Assessment Service for the past 5 years in Korea, lumbar disc herniation (LDH) diagnosed as “Other intervertebral disc disorders (M51.0)” is the fifth highest among musculoskeletal diseases that cause pain. Moreover, LDH was first for 5 consecutive years among patients hospitalized with musculoskeletal diseases. Furthermore, in 2016, the number of patients diagnosed with “Other intervertebral disc disorders (M51.0)” had increased by about 100,000 over the 5 years since 2012. Also, the estimated number of patients receiving Korean Medicine treatment due to other intervertebral disc disorders is considered to be higher than the statistical value of 25,256 because clinically, many patients complaining of back and/or leg pain tend to receive symptomatic treatments without definite diagnoses. Thus, LDH not only makes daily living difficult but also poses a considerable burden on the costs of public healthcare.

In many studies, conservative treatments for LDH have been reported to be effective.^[6,7]^ Among those treatments, acupuncture is in the spotlight to such an extent as to have been upgraded to the recommended level in medical care guidelines.^[8,9]^ Acupotomy, which involves both an acupuncture needle and a surgical scalpel, was developed in 1976 by Professor Han-Zhang Zhu of China. As a modern acupuncture treatment, it has been used to treat patients with pain caused by various diseases and can be used to remove chronic lesions in soft tissue. Some studies done in the Republic of Korea have reported acupotomy to be clinically effective, especially for patients suffering from pain due to LDH.^[10–12]^ However, those studies have all been case studies, so more extensive, well-designed, randomized clinical trials (RCTs) are needed to ensure the effectiveness and safety of acupotomy for treating patients suffering from pain due to LDH.

According to a pilot study implemented by our study team, the reductions in the pain intensity and the disability in the acupotomy group were statistically significantly superior to those in the manual acupuncture group, and acupotomy treatment was found to be as safe as a manual acupuncture treatment. Based on those results, we have planned a large-scale RCT to confirm the effectiveness and safety of acupotomy treatment for patients with back and/or leg pain caused by LDH.

## 2 Methods

### 2.1 Objective

In this multicenter, randomized, controlled, assessor-blinded, clinical trial, we will evaluate the effectiveness and safety of acupotomy for treating back and/or leg pain in patients with LDH. The objectives of this clinical trial are twofold: to compare the pain intensity on the visual analog scale (VAS) in the acupotomy group with that in the manual acupuncture group at 4 weeks after randomization and to investigate adverse events (AE) that occur during the study.

### 2.2 Study design

**Figure 1:**
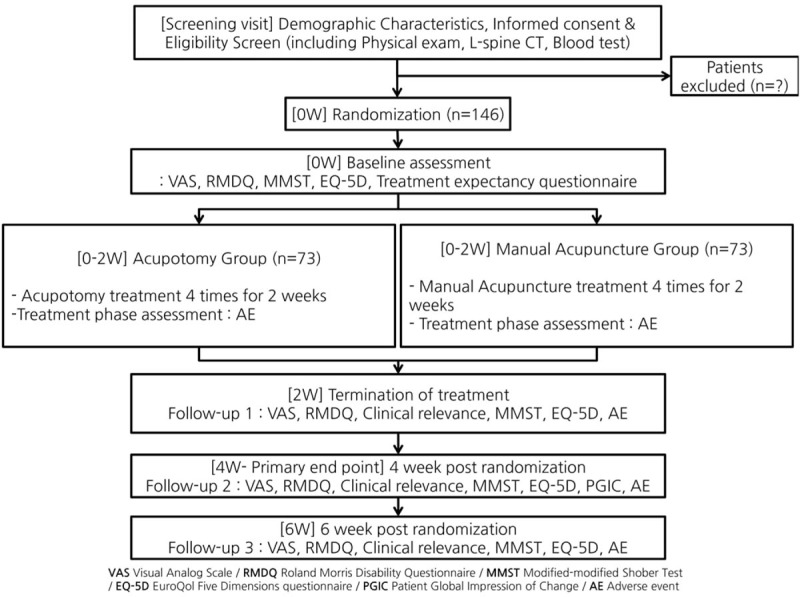
Study flow with outcome assessments.

This study will be performed as a multicenter, 2-arm parallel design, stratified block (male vs female) randomized, assessor-blinded, controlled, clinical trial. The flowchart for this trial is shown in Fig. 1. This study will recruit 73 applicants per group, for a total of 146 applicants, who meet the inclusion and the exclusion criteria (Table 1).^[13]^ A total of 146 patients with LDH will be recruited from outpatients at the Daejeon University Dunsan Korean Medicine Hospital (DUDKMH) and the Daejeon University Cheonan Korean Medicine Hospital through advertisements posted on bulletin boards at subway stations, apartments, and hospitals and on subway cars, hospitals’ homepages, newspapers, etc. Recruitment commenced in March 2018, and the trial is expected to end in February 2019.

**Table 1:**
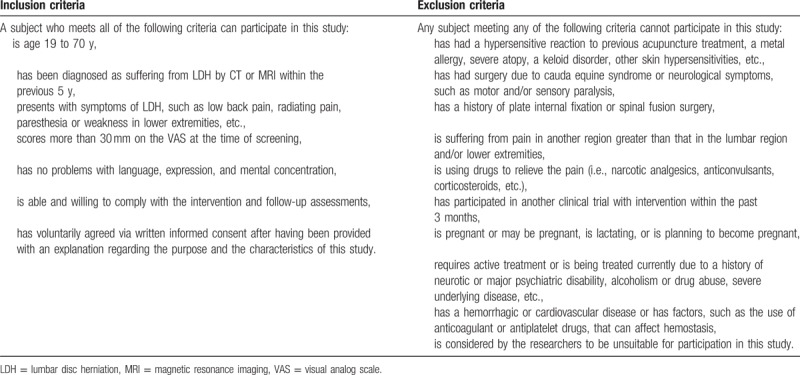
Inclusion and exclusion criteria.

All participants will receive a full explanation of this study's protocol and will be provided a written explanation and informed consent form. Those who have not had computed tomography (CT) or magnetic resonance imaging (MRI) examinations within the past 5 years, but are suspected to have LDH based on history taking or a physical examination, will receive a lumbar spine CT scan. Also, blood tests, such as those for blood clotting factors, liver function, inflammation-related enzymes, and whole blood cells, will be provided. Participants who are judged to be eligible according to criteria for this study will be randomly assigned in a 1:1 ratio to either the acupotomy group or the manual acupuncture group. The intervention will begin within 2 weeks of the screening visit. The subject will receive a total of 4 interventions over 2 weeks, followed by 2 follow-up visits, 1 each at 2 and 4 weeks after the last intervention.

### 2.3 Sample size calculation

Based on the results of a pilot study and clinical significance, we assume that the mean difference (δ) between the changes in the VAS before and after treatment in each group is 15 and the standard deviation (σ) is 24.71. When a significance level of 5%, a power of 90%, and an assignment ratio of the groups of 1:1 are applied, the number of subjects in each group (n) is given by^[14]^ 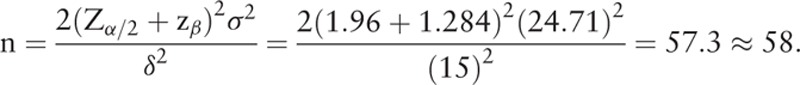


Also, for an expected dropout rate of 20%, according to 
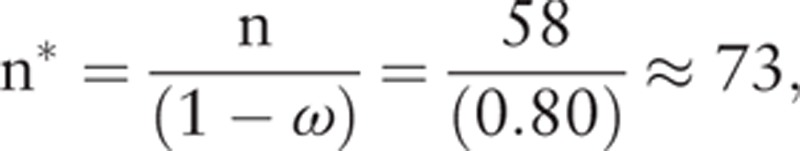


at least 73 subjects per group are required, for a total of 146 subjects.

### 2.4 Randomization and blinding

The randomization will be performed by using a random number table that is generated by using the statistical program SAS Version 9.4 (SAS Institute Inc, Cary, NC) and is kept secret by the statisticians who are independent of this study. Stratified block randomization, which uses gender as a stratification factor, will be performed so that the sex ratio, which is the factor affecting the treatment, will be balanced in the 2 groups.^[15]^

This study is designed so that assessors, data collectors, and statisticians are blinded to the group assignment of the participants. Researchers who do not perform the intervention and randomization will assess the subjects. The assessment items and the contents for the purpose of completing the case report form (CRF) will be simply discussed with minimal dialogue, but will be closely evaluated. Furthermore, the study will be conducted in such a manner that the researchers will not know what intervention the subject is receiving. However, in this study, the 2 interventions are so different that blinding the clinicians and the subjects will be impossible. Thus, the clinicians of acupotomy and those of manual acupuncture will be separated from each other, and each clinician will perform only 1 kind of intervention; this should prevent biases due to intentional intervention by the clinicians. Moreover, the intervention clinicians will not participate in any assessment.

### 2.5 Intervention

#### 2.5.1 Acupotomy

**Figure 2:**
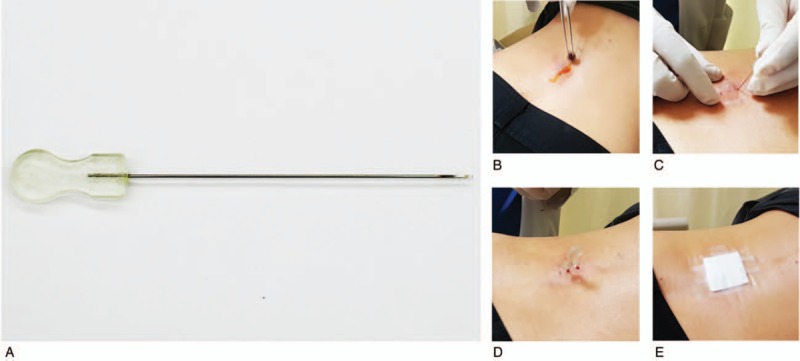
Application of acupotomy: (A) acupotomy needle, (B) skin disinfection, (C) needle insertion, (D) needle manipulation, and (E) disinfection and dressing.

The patients in the acupotomy group will receive a total of 4 acupotomy treatments over 2 weeks (Fig. 2). The needle for acupotomy (DongBang Acupuncture Inc, Chungcheongnam-do, Republic of Korea) with a circumferential shape, a diameter of 0.75 mm, and a length of 80 mm will be used. Before intervention, the clinicians will check the level of the herniated lumbar disc that has been diagnosed via a lumbar spine CT or MRI scan. When the subject is in the prone position, 2 to 6 points of the inner core muscles, which are placed bilaterally 20 to 30 mm from the spinous process of the level of the herniated lumbar vertebral disc and the soft tissue, which feels stiff and hard, will be selected by the clinicians. After the lumbar region has been sterilized with cotton saturated with 78% alcohol, the clinicians will take 1 to 2 g of emla cream 5% (AstraZeneca Korea, lidocaine and prilocaine, each 25 mg per 1 g) and spread it thinly on those 2 to 6 points and their environs. Then, this region will be covered and sealed with plastic wrap to block air so that it will be anesthetized. Next, that region will be sterilized using a cotton pad soaked with a 10% povidone-iodine solution. At the selected points, the 2 to 6 needles for acupotomy will be inserted to a depth of 50 to 70 mm, manipulated so as to alleviate the induration through incision and exfoliation, and then pulled out immediately without retention.^[16]^ Finally, the treatment areas will be sterilized and dressed with gauze. The methods for preventing infection at the treatment site will be carefully explained to all patients in the acupotomy group. All acupotomy treatments will be performed by Korean Medicine doctors who have had acupotomy treatment experience for more than 2 years. (See Appendix 1, Supplemental Content, which illustrates the STRICTA, Standards for Reporting Interventions in Clinical Trials of Acupuncture)

#### 2.5.2 Manual acupuncture

The patients in the manual acupuncture group will receive a total of 4 manual acupuncture treatments over 2 weeks as a control group. In this trial, 0.25 × 40 mm disposable sterile stainless-steel needles (DongBang Acupuncture Inc, Chungcheongnam-do, Republic of Korea) will be used. First, with the subject in the prone position, the lumbar region will be sterilized using cotton saturated with 78% alcohol. The acupuncture treatment will be performed on the local GV3 (Yaoyangguan) point, the bilateral BL23 (Shenshu), BL24 (Qihaishu), BL25 (Dachangshu), and BL26 (Guanyuanshu) points, and the distant bilateral GB30 (Huantiao), BL40 (Weizhong), and BL60 (Kunlun) points. For inducing Deqi sensation, we will rotate the needles left and right 3 to 5 times and then keep the needles in place for 15 minutes.^[17]^ After the needle has been removed, the treatment area will be disinfected using cotton saturated with 78% alcohol. All manual acupuncture treatments will be performed by Korean Medicine doctors who have had manual acupuncture treatment experience for more than 2 years. For compensation, after the trial is completed, the subjects in the manual acupuncture group will receive a maximum of 2 sessions of acupotomy treatment, upon request.

#### 2.5.3 Cointerventions

All subjects will be instructed not to receive any treatment for back and/or leg pain, except for the treatments we provide. During the period of participation in the clinical trial, they will receive rescue medication (i.e., acetaminophen, up to 3000 mg per day). They will be instructed to take the rescue medicine only when their back and/or leg pain is so severe that it is unbearable. At each visit, the clinical research coordinator (CRC) will accurately record on the CRF the total rescue medication dosage used by the subject since the last visit.

At the discretion of the researching physician, all subjects will be allowed to continue to take medications that they were taking before their participation in this study. The subjects will be instructed to report to the researcher at each visit any changes in the dosages of the medications that they have been taking or any new medications that they have taken since they began participating in this study. They will also be instructed not to take arbitrarily a drug that might affect the study's outcomes. If the subject does not follow the above instructions and if the principal investigator (PI) judges that those actions could significantly influence the study's outcomes, that subject will be dropped from this study.

### 2.6 Outcome measures

Assessments will be conducted at baseline and at 2 weeks (2W), 4 weeks (4W), and 6 weeks (6W) after randomization. The assessments at baseline and 2W during the intervention period will be performed before the assigned intervention for that visit. The schedule for the assessments of the results is shown in Table 2.

**Table 2:**
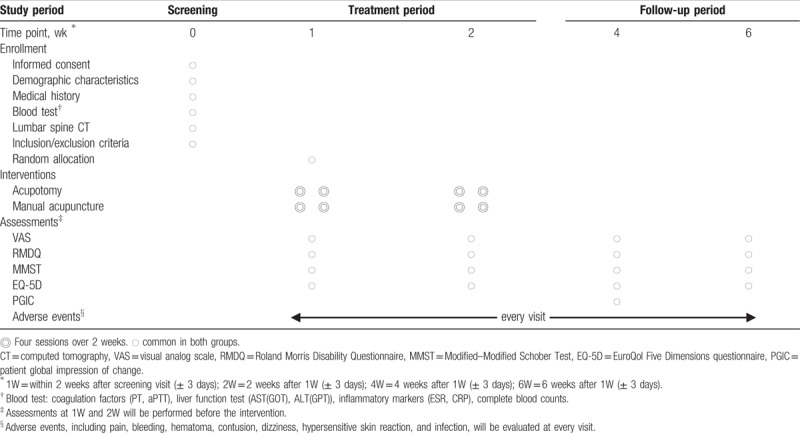
Schedule for the treatment and the outcome measurements.

The primary outcome is the mean change in the VAS score for back and/or leg pain at 4W compared with baseline. The baseline VAS score will be measured immediately before the first intervention. The subject will indicate the degree of back and/or leg pain on a straight line of 100 mm. On that line, the left endpoint, 0, is no pain, and the right endpoint, 100, is the maximum pain imaginable.^[18]^

The secondary outcomes include back and/or leg pain at other time points, the clinical relevance^[19–22]^, dysfunction of the lumbar spine, range of motion of the lumbar spine, quality of life, and global assessment.

The clinical relevance will be assessed by comparing the percentage of subjects who indicate an improvement of more than 15 mm on the VAS, which corresponds to the Minimal Clinically Important Difference (MCID), of more than 30%, and of more than 50% between the 2 groups. In 2008, a consensus statement was issued that recommended MCID values for chronic pain assessment tools (pain, dysfunction, psychological factors, quality of life, etc.). According to a study that investigated the clinical relevance of the VAS for chronic back pain, the MCID was found to be greater than 15 mm based on the VAS (0–100 mm). Thus, in this study, we compare the proportions of subjects who have a decrease in the VAS score of 15 mm in each of the 2 groups. According to the above recommendation, in assessments using the VAS or the NRS, reductions of more than 30% and 50% in the MCID in chronic pain research suggest moderate clinically important changes and substantial improvements, respectively. Therefore, the main assessment tool of this study will be the VAS, so we will use it according to the criteria of the above recommendations.

The dysfunction of the lumbar spine will be assessed by using the mean changes in the scores on the Korean version of the Roland Morris Disability Questionnaire (RMDQ)^[23–26]^ at 2W, 4W, and 6W compared with baseline. The RMDQ was designed to measure the physical dysfunction of patients with back pain. Twenty-four questionnaire items will be simply answered “Yes (1) / No” (0), and the questionnaire will be scored from 0 to 24. A higher score indicates a greater degree of dysfunction. This survey has 2 advantages: the respondents can quickly complete the survey and the survey is easy to apply.

The range of motion of the lumbar spine will be assessed by using the mean changes in the measured values on the Modified-Modified Schober Test (MMST)^[27,28]^ at 2W, 4W, and 6W compared with baseline. The MMST is a physical examination that assesses lumbar vertebrae mobility. With the subject standing straight, the end points 10 cm above and 5 cm below a straight line based on the point where the mid-line of the vertebrae and the connection-line of both Posterior Superior Iliac Spines meet will be marked. Then, the researching physician will let the subject bend his or her lower back forward to the maximum extent while still feeling no pain and will then measure the amount of change in the distance between the 2 points.

The quality of life will be assessed by using the mean changes in the scores on the Korean version of the EuroQol Five Dimensions questionnaire (EQ-5D)^[29,30]^ at 2W, 4W, and 6W compared with baseline. The EQ-5D is the most widely used tool for measuring quality of life. Hence, only the EQ-5D Index will be used in this study.

The global assessment will be done by using the Patient Global Impression of Change (PGIC)^[31]^ at 4W. The PGIC is a method for assessing the alleviation of the symptoms that the subjects have felt themselves before and after treatment. The subjects are able to choose from 7 categories of responses to how much the back and/or leg pain has been reduced compared with before their participation in this study.

### 2.7 Adverse events

In this study, all undesirable medical findings in which symptoms were not observed until participation in this clinical study will be classified as AEs. The researchers will carry out an AE assessment at each visit based on vital signs, history taking, and other test results. In addition, the subjects will be instructed to report voluntarily any adverse situations to the researchers at every visit, and the researcher will confirm such reports through medical examination. All identified AEs will be recorded on the CRF, regardless of their association with acupotomy treatment or manual acupuncture treatment. The researchers will assess the severity of the AE and take appropriate action. If serious adverse events (SAEs) occur, such as a life-threatening event or disorder or any other medically important condition, the PI should decide whether or not the subject should be dropped from this study.

### 2.8 Statistical analysis

The data obtained from the subjects of this study will be analyzed in the form of a full analysis set (FAS) analysis group and a per protocol (PP) analysis group. The FAS analysis group refers to analyzing a set of subjects that minimizes those who are excluded, except for a legitimate reason, from the analysis among the randomly assigned subjects. The PP analysis group refers to analyzing a set of subjects who have participated in more than 75% of the interventions (3 or more interventions) specified in this study's protocol and have not violated this study's protocol, and for whom the assessment variables have been measured completely. Both the FAS analysis group and the PP analysis group will be used for the effectiveness analysis while the main analysis group will be the FAS analysis group and the auxiliary confirmation will be performed by using the PP analysis group.

All statistical analyses will be conducted using the 2-tailed test in principle, and the significance level will be set at 5%. Analyses will use the statistical program SAS Version 9.4 (SAS Institute, Inc, Cary, NC). When missing values are encountered, they will be processed using multiple imputations.

The descriptive statistics of the subjects’ demographic characteristics and clinical baseline variables, such as gender, age, history, and drug dosage, will be presented for each treatment group. If the comparisons between the 2 groups involve continuous data, the mean and the standard deviation will be presented, and analyses will be performed using the independent *t* test or the Wilcoxon rank sum test. As needed, the 95% confidence interval will be provided. If the comparisons between the 2 groups involve categorical data, the frequency and the percentile will be presented, and analyses will be performed using the chi-square test or the Fisher exact test.

The primary outcome variable is the mean changes in the score on the VAS at 4W compared with the baseline and will be analyzed using an analysis of covariance with the covariates being the VAS score at baseline and the fixed factor that in each treatment group. Secondary outcome variables will be analyzed in the same manner as the primary outcome variable. If the outcome variables are categorical data, they will be analyzed using the chi-square test or the Fisher exact test. If necessary, a paired *t* test or Wilcoxon signed-rank test will be used for the primary and the secondary outcome variables to analyze differences in measured values before and after treatment in each group, and a repeated measures analysis of variance will be performed to test the difference in the trend at each visit. The Dunnett procedure will be used for multiple comparisons.

If necessary, subgroup analyses of outcome variables, such as demographic variables measured at baseline (e.g., gender, age, duration of illness, treatment expectancy, etc.), whether or not rescue medicines were taken, the amounts of rescue medications taken, the hospital at which the subject was treated, etc., may be conducted. The safety assessments will primarily be performed by analyzing the frequency of adverse events and SAEs that the researchers suspect to be associated with treatment. The collected safety data will be appropriately summarized. All adverse events that occur will be listed with a detailed description. All SAEs will be recorded descriptively. Adverse events will be collected through patient symptom reports and researcher observations. The frequency of each adverse event associated or not associated with intervention will be recorded and analyzed using descriptive statistics. No interim analysis will be performed.

### 2.9 Withdrawal and dropout

If any subject withdraws consent, is found to be in violation of the inclusion and exclusion criteria, etc., or if the PI judges that continuing participation of the subject in this study is inappropriate, he or she will be dropped from this study. The researchers will record whether each subject participating in this study completed it or not, as well as the reason for any interruption in the intervention.

### 2.10 Ethics and monitoring

This study is designed based on the Helsinki Declaration and the Korean Clinical Practice Guidelines and has been approved by the Korean Institutional Review Board of DUDKMH (number DJDSKH-18-BM-02). This study protocol is registered with the Korean National Clinical Research Information Service (WHO) (CRIS-KCT0002824). All subjects participating in this study may withdraw their consent at any time for any reason and may discontinue their participation on a voluntary basis.

The data and records related to the conduct of the clinical research will be maintained in separate places at DUDKMH and Daejeon University Cheonan Korean Medicine Hospital. At the completion of the study, independent researchers will edit, classify, and code data for statistical analyses. The monitoring of data and research performances will be conducted regularly by researchers from the Korea Institute of Oriental Medicine. The results of this study will be published in a peer-reviewed paper.

## 3 Discussion

This study is designed to compare the clinical effectiveness of acupotomy treatment with that of manual acupuncture treatment for patients suffering from lower back or leg pain due to LDH. Reductions in pain and dysfunction and increases in lumbar mobility and quality of life will be evaluated to confirm whether the subjects with LDH had improved or not. In addition, a safety assessment will be conducted by examining any adverse events that might occur in any of the participants in this study.

LDH may cause back and/or leg pain due to physical stress on the herniated area, chemical (inflammatory) stimuli, microcirculatory circulation disturbance, edema of the nerve root region, and the like.^[32,33]^ As shown in other studies, acupotomy treatment is effective in reducing back and/or leg pain because it relieves chronic adhesions and blood flow disturbances around the nerve by using a needle thicker than that of manual acupuncture to stimulate strongly the local area.^[16,34]^ The authors of this study have applied acupotomy treatment to patients with a variety of vertebral diseases and have published a number of case reports^[10–12]^ and a pilot study.^[14]^ The results of the pilot study confirmed, to some extent, the clinical effectiveness and safety of the acupotomy treatment, but the study had some limitations. First, it was a single center study. Second, patients in the acupotomy group received a lidocaine injection before acupotomy treatment and a wet-cupping treatment after acupotomy treatment, but patients in the manual acupuncture group did not. This is because the pilot study compared the 2 acupuncture treatments in the same manner as they are used in clinical practice. Finally, the pilot study had only 1 follow-up assessment, which occurred at 4 weeks after randomization. Therefore, to minimize these limitations, we designed this study as a large-scale, multicenter study. In addition, neither lidocaine injections nor wet-cupping treatments were used in either group. This is because this study will compare 2 acupuncture treatments in such a way that the numbers and frequencies of those treatments are the same; only the stimulation method is different. When the acupotomy needle is inserted, however, the pain at the treated site is so severe that the skin at that site needs to be anesthetized; in this study, only lidocaine cream was used for that purpose. In addition, for longer and more accurate observation, this study includes 2 follow-up assessments, 1 at 4 weeks and the other at 6 weeks after randomization.

This study has some strengths. The researchers diagnose the LDH of the subjects by using comprehensive judgments based on radiologic examination (CT or MRI), physical examination, clinical symptoms (back and/or leg pain), medical opinion of the clinician, etc., and then judge whether or not he/she can participate in this study. In addition, blood tests and history taking, which are conducted at the screening visit, can be used to exclude subjects with hemostatic disorders, which should prevent SAEs that may occur as a result of the acupotomy treatment.

This study, however, also has some weaknesses. The results are examined up to 6 weeks after randomization (6W), which is still a rather short follow-up period. Also, some differences may exist between the 2 groups in the treatment method of intervention (e.g., treated site, depth of needle insertion, total amount of stimulation, etc.). Because the procedures of the 2 interventions are so different, neither the practitioner nor the patients could be blinded, and the presence of nonspecific variables (e.g., expectation of the subject about the clinicians and the interventions of this study, the subject's expectation on the alleviation of the symptoms through the acupotomy treatment, threshold of pain, etc.) cannot be excluded. Nevertheless, because of the multicenter RCT design of this study, the results are expected to provide useful information for confirming the effectiveness of acupotomy for the treatment of patients with LDH symptoms. Furthermore, the results are expected to confirm that acupotomy treatment for such patients is as safe as manual acupuncture treatment.

## Author contributions

JKJ designed the study and drafted the manuscript. YIK and C-HH participated in the design of the study as principal investigators and were responsible for the final decision to submit this manuscript for publication. EK, HJK, and KSY participated in the design of the study. JHJ, JHK, and OK developed the plan for the statistical analysis. HL and S-YJ provided technical advice and made critical revisions for the study plan and the manuscript. All authors read and approved the final manuscript.

**Conceptualization:** Jeong Kyo Jeong, Young Il Kim.

**Data curation:** Ojin Kwon, So-Young Jung.

**Formal analysis:** Ojin Kwon.

**Funding acquisition:** Chang-Hyun Han.

**Investigation:** Jeong Kyo Jeong, Hae Jin Kong.

**Methodology:** Eunseok Kim, Kwang Sik Yoon, Ju Hyun Jeon, Jae Hui Kang.

**Project administration:** Jae Hui Kang, Hyun Lee, So-Young Jung.

**Software:** Ojin Kwon.

**Supervision:** Young Il Kim, Chang-Hyun Han.

**Validation:** Hyun Lee, So-Young Jung.

**Writing – original draft:** Jeong Kyo Jeong.

**Writing – review & editing:** Eunseok Kim.

## Supplementary Material

Supplemental Digital Content
