# Supplementary material for: Effectiveness and safety of acupotomy for treating back and/or leg pain in patients with lumbar disc herniation: A study protocol for a multicenter, randomized, controlled, clinical trial
Source: Medicine (Baltimore). 2018 Aug 24;97(34):e11951. doi: 10.1097/MD.0000000000011951 (PMC6112943; doi:10.1097/MD.0000000000011951)
Supplement: Supplemental Digital Content [file medi-97-e11951-s001.pdf]

Appendix 1. STRICTA (Standards for Reporting Interventions in Clinical Trials of Acupuncture)

Item		Detail	Answer
1. Acupuncture rationale	1a)	Style of acupuncture (e.g. Traditional Chinese Medicine, Korean, Japanese, Western medical, Five Element, ear acupuncture, etc.)	Acuptomy
	1b)	Reasoning for treatment provided, based on historical context, literature sources, and/or consensus methods, with references where appropriate	Textbook*<br>Published articles†
	1c)	Extent to which treatment was varied	Personalized treatment that the patient's inner core muscle around the level of herniated lumbar vertebral disc and the soft tissue where stiffened and hardened parts are palpable treated will be performed.
2. Details of needling	2a)	Number of needle insertions per subject per session (mean and range where relevant)	2~6 points per subject
	2b)	Names (or location if no standard name) of points used (uni/bilateral)	Bilateral points that are parallel to the left and right 20-30 mm apart from spinous process of lumbar spine
	2c)	Depth of insertion, based on a specified unit of measurement, or on a particular tissue level	50-70 mm, Deep muscles of intervertebral region that stiffened and hardened parts are palpable
	2d)	Response sought (e.g. de qi or muscle twitch response)	Local pain and twinge like de qi sensation (penetrating, sharp, aching and painful sensations when penetrating the skin, spreading and lumpish sensation around the acupuncture site)
	2e)	Needle stimulation (e.g. manual, electrical)	Manipulation
	2f)	Needle retention time	After insertion and manipulation, needle will be pulled out immediately without retention
	2g)	Needle type (diameter, length, and manufacturer or material)	Sterilized disposable acupuncture (DongBang Acupuncture Inc., Chungcheongnam-do, Republic of Korea) 0.75 mm×80 mm
3. Treatment regimen	3a)	Number of treatment sessions	4 times

	3b)	Frequency and duration of treatment sessions	4 times per two weeks
4. Other components of treatment	4a)	Details of other interventions administered to the acupuncture group(e.g. moxibustion, cupping, herbs, exercises, lifestyle advice)	Not done
	4b)	Setting and context of treatment, including instructions to practitioners, and information and explanations to patients.	If the participants are selected as the subject of the clinical trial at the screening visit, they would visit the clinical research center four times and receive the acupotomy treatment.
5. Practitioner background	5)	Description of participating acupuncturists (qualification or professional affiliation, years in acupuncture practice, other relevant experience)	Korean Medicine Doctors (KMD) who has a more than two years' career of acupotomy and is licensed by Ministry of Health and Welfare, Korea.
6. Control or comparator interventions	6a)	Rationale for the control or comparator in the context of the research question, with sources that justify this choice.	
	•	The points treated by acupuncture	Textbook‡
	•	General effect about lumbago treated by acupuncture as controlled group	Published articles§

•	The number of treatment sessions of controlled group compared with that of experimental group	Generally, acupotomy treatment is performed twice or three times a week and manual acupuncture treatment is performed twice or four times a week at lumbar disc herniation treatment.In the clinical, total number of treatment sessions is different individually depending on patients' state and symptoms. On this clinical trial, however, the total number of treatment sessions would be controlled equally as four times a week, so that effectiveness comparison between two interventions would be expected to be clear.For example, on the study about additional effectiveness of acupotomy when manual acupuncture treatment combined with acupotomy treatment is performed, the trial was set that experimental group received ten times' manual acupuncture treatment and once or twice acupotomy treatment and controlled group received only ten times' manual acupuncture treatment. ‖ On the another study, the number of treatment sessions of pharmacopuncture treatment as experimental group compared with that of manual acupuncture treatment as controlled group was set equally.¶
6b)	Precise description of the control or comparator.<br>If sham acupuncture or any other type of acupuncture-like control is used, provide details as for Items 1 to 3 above.	
(1a)	Style of acupuncture(e.g. Traditional Chinese Medicine, Japanese, Korean, Western medical, Five Element, ear acupuncture, etc)	Manual acupuncture

(1b)	Reasoning for treatment provided, based on historical context, literature sources, and/or consensus methods, with references where appropriate	Textbook‡<br>Published articles§
(1c)	Extent to which treatment was varied	Within 15 predefined acupuncture points
(2a)	Number of needle insertions per subject per session (mean and range where relevant)	15
(2b)	Names (or location if no standard name) of points used (uni/bilateral)	On the local points, GV3 (Yaoyangguan) and bilateral BL23 (Shenshu), BL24 (Qihaishu), BL25 (Dachangshu), and BL26 (Guanyuanshu).<br>On the distant points, the bilateral GB30 (Huantiao), BL40 (Weizhong), and BL60 (Kunlun).
(2c)	Depth of insertion, based on a specified unit of measurement, or on a particular tissue level	30 mm at GV3 (Yaoyangguan) and bilateral BL23 (Shenshu), BL24 (Qihaishu), BL25 (Dachangshu), BL26 (Guanyuanshu) and GB30 (Huantiao).<br>20 mm at the bilateral BL40 (Weizhong), and BL60 (Kunlun).
(2d)	Response sought (e.g. de qi or muscle twitch response)	De qi sensation (penetrating, sharp, aching and painful sensations when penetrating the skin, spreading and lumpish sensation around the acupuncture site)
(2e)	Needle stimulation (e.g. manual, electrical)	Manual stimulation
(2f)	Needle retention time	15 minutes
(2g)	Needle type (diameter, length, and manufacturer or material)	0.25×40 mm disposable sterile stainless steel acupuncture needles (DongBang Acupuncture Inc., Chungcheongnam-do, Republic of Korea)
(3a)	Number of treatment sessions	4 times
(3b)	Frequency and duration of treatment sessions	4 times per two weeks

*Korean Acupuncture & Moxibution Soc. The Acupunct Acupuncture and Moxibution Medicine. Paju: Hanmi medical publishing co. 2016: 173-80.

†Kim JH, Jang YJ, Park JH, You YN. Clinical Research: The Effects of Korean Medical Treatment Combined with Acupotomy on Patients with a Herniated Intervertebral Disc of the Lumbar Spine: a Retrospective Study. The Acupunct. 2015 Mar: 32(1); 119-26.

‡Korean Acupuncture & Moxibution Soc. The Acupunct Acupuncture and Moxibution Medicine. Paju: Hanmi medical publishing co. 2016: 510-3.

§Andrea D Furlan, Maurits W van Tulder, Dan Cherkin. Acupuncture and dry-needling for low back pain. Cochrane Database of Systematic Reviews. 2005; DOI: 10.1002/14651858.CD001351.pub2.

‖ Yun JY, Kim DH, Kim HW, Kim SS, Park SW, Kim EK, Lee GH, Lee GM. The Clinical Effects of Acupuncture and Acupotomy Therapy for HIVD. J Korean Acupunct Moxib Soc. 2010 Aug: 27(4); 85-97.

¶Lee AR, Kim WI. The Retrospective Comparative Study of General Acupuncture Therapy and Hominis placenta Pharmacopuncture Therapy on Severe Dyspepsia. Korean Journal of Acupuncture. 2013 Dec; 30(4): 319-28.

Kang I, Moon JY, Lim MJ, Cho JH, Lee HE. The Comparison Study between Different Interventions for Treating Acute Ankle Sprain. J Korean Acupunct Moxib Soc. 2008 Oct; 25(5): 89-95.

Park KB, Song KH, Lee JS, Jo JH. Study on Clinical Effects of Homnis Placenta Herbal Acupuncture on Osteoarthritis of Knee Joint. J Korean Acupunct Moxib Soc. 2006 Aug; 23(4): 163-73.

Lee JH, Shin JS, Lee YJ, Kim MR, Ahn YJ, Park KB, Michael A. Kropf, Shin BC, Lee MS, Ha IH. Effects of Shinbaro pharmacopuncture in sciatic pain patients with lumbar disc herniation: study protocol for a randomized controlled trial. Trials. 2015 Oct; DOI: 10.1186/s13063-015-0993-6.

STANDARD PROTOCOL ITEMS: RECOMMENDATIONS FOR INTERVENTIONAL TRIALS

SPIRIT 2013 Checklist: Recommended items to address in a clinical trial protocol and related documents*

Section/item	Item No	Description	Addressed on page number
**Administrative information**			
Title	1	Descriptive title identifying the study design, population, interventions, and, if applicable, trial acronym	Title page
Trial registration	2a	Trial identifier and registry name. If not yet registered, name of intended registry	Abstract & 9
	2b	All items from the World Health Organization Trial Registration Data Set	9
Protocol version	3	Date and version identifier	Not Applicable
Funding	4	Sources and types of financial, material, and other support	9
Roles and responsibilities	5a	Names, affiliations, and roles of protocol contributors	9
	5b	Name and contact information for the trial sponsor	Title page & 9
	5c	Role of study sponsor and funders, if any, in study design; collection, management, analysis, and interpretation of data; writing of the report; and the decision to submit the report for publication, including whether they will have ultimate authority over any of these activities	9
	5d	Composition, roles, and responsibilities of the coordinating centre, steering committee, endpoint adjudication committee, data management team, and other individuals or groups overseeing the trial, if applicable (see Item 21a for data monitoring committee)	Not Applicable

Introduction

Background and rationale	6a	Description of research question and justification for undertaking the trial, including summary of relevant studies (published and unpublished) examining benefits and harms for each intervention	1-2
	6b	Explanation for choice of comparators	1-3
Objectives	7	Specific objectives or hypotheses	1, 7-8
Trial design	8	Description of trial design including type of trial (eg, parallel group, crossover, factorial, single group), allocation ratio, and framework (eg, superiority, equivalence, noninferiority, exploratory)	1-2

Methods: Participants, interventions, and outcomes

Study setting	9	Description of study settings (eg, community clinic, academic hospital) and list of countries where data will be collected. Reference to where list of study sites can be obtained	2
Eligibility criteria	10	Inclusion and exclusion criteria for participants. If applicable, eligibility criteria for study centres and individuals who will perform the interventions (eg, surgeons, psychotherapists)	Table 1 & 2-4
Interventions	11a	Interventions for each group with sufficient detail to allow replication, including how and when they will be administered	3-4
	11b	Criteria for discontinuing or modifying allocated interventions for a given trial participant (eg, drug dose change in response to harms, participant request, or improving/worsening disease)	5, 7
	11c	Strategies to improve adherence to intervention protocols, and any procedures for monitoring adherence (eg, drug tablet return, laboratory tests)	3-5
	11d	Relevant concomitant care and interventions that are permitted or prohibited during the trial	4
Outcomes	12	Primary, secondary, and other outcomes, including the specific measurement variable (eg, systolic blood pressure), analysis metric (eg, change from baseline, final value, time to event), method of aggregation (eg, median, proportion), and time point for each outcome. Explanation of the clinical relevance of chosen efficacy and harm outcomes is strongly recommended	4-6
Participant timeline	13	Time schedule of enrolment, interventions (including any run-ins and washouts), assessments, and visits for participants. A schematic diagram is highly recommended (see Figure)	4 & Table 2

Sample size	14	Estimated number of participants needed to achieve study objectives and how it was determined, including clinical and statistical assumptions supporting any sample size calculations	2
Recruitment	15	Strategies for achieving adequate participant enrolment to reach target sample size	2

Methods: Assignment of interventions (for controlled trials)

Allocation:			
Sequence generation	16a	Method of generating the allocation sequence (eg, computer-generated random numbers), and list of any factors for stratification. To reduce predictability of a random sequence, details of any planned restriction (eg, blocking) should be provided in a separate document that is unavailable to those who enrol participants or assign interventions	2-3
Allocation concealment mechanism	16b	Mechanism of implementing the allocation sequence (eg, central telephone; sequentially numbered, opaque, sealed envelopes), describing any steps to conceal the sequence until interventions are assigned	2-3
Implementation	16c	Who will generate the allocation sequence, who will enrol participants, and who will assign participants to interventions	2-3
Blinding (masking)	17a	Who will be blinded after assignment to interventions (eg, trial participants, care providers, outcome assessors, data analysts), and how	2-3
	17b	If blinded, circumstances under which unblinding is permissible, and procedure for revealing a participant's allocated intervention during the trial	5

Methods: Data collection, management, and analysis

Data collection methods	18a	Plans for assessment and collection of outcome, baseline, and other trial data, including any related processes to promote data quality (eg, duplicate measurements, training of assessors) and a description of study instruments (eg, questionnaires, laboratory tests) along with their reliability and validity, if known. Reference to where data collection forms can be found, if not in the protocol	4-5
	18b	Plans to promote participant retention and complete follow-up, including list of any outcome data to be collected for participants who discontinue or deviate from intervention protocols	3-4

Data management	19	Plans for data entry, coding, security, and storage, including any related processes to promote data quality (eg, double data entry; range checks for data values). Reference to where details of data management procedures can be found, if not in the protocol	7
Statistical methods	20a	Statistical methods for analysing primary and secondary outcomes. Reference to where other details of the statistical analysis plan can be found, if not in the protocol	6
	20b	Methods for any additional analyses (eg, subgroup and adjusted analyses)	6
	20c	Definition of analysis population relating to protocol non-adherence (eg, as randomised analysis), and any statistical methods to handle missing data (eg, multiple imputation)	6
**Methods: Monitoring**			
Data monitoring	21a	Composition of data monitoring committee (DMC); summary of its role and reporting structure; statement of whether it is independent from the sponsor and competing interests; and reference to where further details about its charter can be found, if not in the protocol. Alternatively, an explanation of why a DMC is not needed	7
	21b	Description of any interim analyses and stopping guidelines, including who will have access to these interim results and make the final decision to terminate the trial	6
Harms	22	Plans for collecting, assessing, reporting, and managing solicited and spontaneously reported adverse events and other unintended effects of trial interventions or trial conduct	5
Auditing	23	Frequency and procedures for auditing trial conduct, if any, and whether the process will be independent from investigators and the sponsor	Not Applicable
**Ethics and dissemination**			
Research ethics approval	24	Plans for seeking research ethics committee/institutional review board (REC/IRB) approval	7, 9
Protocol amendments	25	Plans for communicating important protocol modifications (eg, changes to eligibility criteria, outcomes, analyses) to relevant parties (eg, investigators, REC/IRBs, trial participants, trial registries, journals, regulators)	Not Applicable

Consent or assent	26a	Who will obtain informed consent or assent from potential trial participants or authorised surrogates, and how (see Item 32)	2, 9
	26b	Additional consent provisions for collection and use of participant data and biological specimens in ancillary studies, if applicable	Not Applicable
Confidentiality	27	How personal information about potential and enrolled participants will be collected, shared, and maintained in order to protect confidentiality before, during, and after the trial	2-3, 7
Declaration of interests	28	Financial and other competing interests for principal investigators for the overall trial and each study site	9
Access to data	29	Statement of who will have access to the final trial dataset, and disclosure of contractual agreements that limit such access for investigators	7
Ancillary and post-trial care	30	Provisions, if any, for ancillary and post-trial care, and for compensation to those who suffer harm from trial participation	4
Dissemination policy	31a	Plans for investigators and sponsor to communicate trial results to participants, healthcare professionals, the public, and other relevant groups (eg, via publication, reporting in results databases, or other data sharing arrangements), including any publication restrictions	9
	31b	Authorship eligibility guidelines and any intended use of professional writers	Not Applicable
	31c	Plans, if any, for granting public access to the full protocol, participant-level dataset, and statistical code	Not Applicable
**Appendices**			
Informed consent materials	32	Model consent form and other related documentation given to participants and authorised surrogates	Not Applicable
Biological specimens	33	Plans for collection, laboratory evaluation, and storage of biological specimens for genetic or molecular analysis in the current trial and for future use in ancillary studies, if applicable	Not Applicable

*It is strongly recommended that this checklist be read in conjunction with the SPIRIT 2013 Explanation & Elaboration for important clarification on the items. Amendments to the protocol should be tracked and dated. The SPIRIT checklist is copyrighted by the SPIRIT Group under the Creative Commons "Attribution-NonCommercial-NoDerivs 3.0 Unported" license.
